# Visual Targets Aren’t Irreversibly Converted to Motor Coordinates: Eye-Centered Updating of Visuospatial Memory in Online Reach Control

**DOI:** 10.1371/journal.pone.0092455

**Published:** 2014-03-18

**Authors:** Aidan A. Thompson, Patrick A. Byrne, Denise Y. P. Henriques

**Affiliations:** 1 Centre for Vision Research, York University, Toronto, Ontario, Canada; 2 School of Kinesiology & Health Science, York University, Toronto, Ontario, Canada; University of Texas at San Antonio, United States of America

## Abstract

Counter to current and widely accepted hypotheses that sensorimotor transformations involve converting target locations in spatial memory from an eye-fixed reference frame into a more stable motor-based reference frame, we show that this is not strictly the case. Eye-centered representations continue to dominate reach control even during movement execution; the eye-centered target representation persists after conversion to a motor-based frame and is continuously updated as the eyes move during reach, and is used to modify the reach plan accordingly during online control. While reaches are known to be adjusted online when targets *physically* shift, our results are the first to show that similar adjustments occur in response to changes in *representations* of remembered target locations. Specifically, we find that shifts in gaze direction, which produce predictable changes in the internal (specifically eye-centered) representation of remembered target locations also produce mid-transport changes in reach kinematics. This indicates that representations of remembered reach targets (and visuospatial memory in general) continue to be updated relative to gaze even after reach onset. Thus, online motor control is influenced dynamically by both the external and internal updating mechanisms.

## Introduction

It has been found previously that reach movements are modified mid-transport to compensate for unexpected shifts of an actual physical target location (e.g., [1]), but it is unknown if reach trajectories are similarly modified in response to shifts in the internal eye-centered representation of targets when the eyes move after reach onset. Here we show that reaches are indeed adjusted online when *internal representations* of remembered target locations change during the reach, just as when targets shift *physically*.

Previous electrophysiological and behavioral studies have demonstrated that internal representations of movement goals are remapped with eye movements [2]–[10], at least prior to reach onset. This can be seen behaviorally via the retinal magnification effect [11]. This systematic pattern is characterized by reaching errors that exaggerate the retinal eccentricity of remembered target locations. That is, when people look away from a previously displayed target before reaching to the remembered location of that target, final rightward gaze directions (relative to the remembered target locations) result in leftward pointing errors and *vice versa*. Notably, the direction of reach error depends only on target-relative gaze location at the time of reaching, *not* at the time of encoding, which can only be explained if the representation of target location is constantly updated in a gaze-centered frame of reference. This interpretation is consistent with numerous findings from other studies demonstrating eye-centered remapping prior to reach onset (e.g., [4]–[10], [12]–[14]).

Eye-centered representations must necessarily be converted to more stable (likely multiple) reference frames for action [15]–[22]. Here we investigate if the conversion to motor-based reference frames prior to reaching (Cf., the “conversion on demand model [4]) is a permanent or complete conversion, or if the original eye-centered representation persists beyond reach onset (i.e., *during* reaching). If an eye-centered representation persists beyond reach onset, then a mid-reach saccade should remap target *representations*, presumably leading to mid-transport trajectory modification like when targets shift *physically*. In this case, reaches should systematically overshoot the target site as a function of the new final target-relative gaze direction (FGD), and thus resemble the trajectories and errors produced when gaze is at this same FGD prior to reach initiation. Again, if this internal representation is updated, it is updated as the result of a seemingly irrelevant eye movement, and not based on any change to the physical world or sensory information related to the target. Alternatively, if target locations are converted exclusively to some motor-based frame before movement initiation as typically assumed, reaches should systematically overshoot as a function of only *initial* target-relative gaze direction (IGD) like our control trials where the eyes remain fixed throughout the reach.

## Materials and Methods

### Ethics statement

These experiments were approved by the Human Participants Review Sub-committee of York University’s Research Ethics Board. All participants provided written informed consent, and were treated in accordance with the ethical guidelines of York University’s Human Participants Review Sub-committee.

### Participants

Our first experiment consisted of four separate conditions: *Pre-onset saccade, Post-onset saccade, Onset saccade*, and *No saccade control conditions.* The *Pre-onset saccade* and *Onset saccade conditions* had nine healthy right handed participants (5 male, 4 female). The *Post-onset saccade condition* also included nine healthy right handed participants (5 male, 4 female), but only two of the same participants as the *Pre-onset saccade* and *Onset saccade conditions* (because the *Post-onset saccade condition* was collected later, 7 of the original participants were no longer available for testing, and were replaced with gender matched participants of similar age). The *No saccade control condition*, included four participants (1 male, 3 female; one of whom had participated in all conditions, and three of whom had participated in only the *Post-onset saccade condition*). Our follow-up experiment, the *Target representation reliability experiment* included eight participants (5 male; 3 female; one of whom had participated in all previous conditions, and seven of whom had not previously participated). All were between 20 – 30 years of age, had normal or corrected-to-normal vision, were recruited by word of mouth, and received no compensation for their participation in the study.

### Apparatus

Eye movements of the right eye were recorded by infrared pupil identification with the EyeLinkII eye tracker (SR Research Ltd., Osgoode, ON). The left eye, which was not recorded, was patched to ensure that their pointing was based on vision from the recorded eye (Cf., [14], [23], [24]). The three-dimensional position of the head, upper arm, and fingertip were recorded using the OPTOTRAK Certus (Northern Digital Inc., Waterloo, ON) 3D motion capture system. All calibrations and measurement parameters (of both systems), and IRED placements were identical to those from our previous experiments [14]. Recordings from the EyeLinkII and the OPTOTRAK were simultaneously controlled by The MotionMonitor (Innovative Sports Training, Chicago, IL), ensuring a com­mon temporal and spatial reference between the two data sets.

### Stimuli

All visual stimuli were generated by an Optikon XYLP-C Laser Projector (Optikon Corporation Ltd., Kitchener, ON) and rear projected onto a 178 cm matte display surface situated 150 cm from the participants’ eyes. The stimuli used in the study consisted of an array of fixation-crosses and pointing targets (diamonds). Diamonds spanned 1.25 cm or 0.48°^­^of visual angle, while the crosses spanned 2 cm or 0.76°. The center pointing target (diamond) was located in line with the participant’s right eye, while the other two targets were located 5° (or 13.02 cm) to its left and right. Crosses were located 0°, 5°, and 10°both left and right of center. All stimuli were at the same elevation as the eye at a viewing distance of 1.5 m.

### Experimental setup

In each of the conditions described below participants pointed to the remembered location of a briefly flashed target in complete darkness with a fully extended index finger and arm, after moving their eyes in some eccentric direction. Participants were seated at a table, with their heads fixed on a rigidly-mounted, personalized dental impression. Each trial began with participants pressing down on a single button mouse (Apple Canada Inc., Markham, ON) located to the right of the body and within comfortable reach (i.e., aligned with the shoulder for neutral resting position). The button press was used as a release switch for the display (targets only appeared when participants had their reaching hand at the start position) marked movement onset (the release of the mouse) and the end of the trial (the return to the mouse). If the mouse was released at the wrong time (during the target display), that is if participants moved their hand too soon, the trial was aborted and repeated at a later time. To prevent dark adaptation a halogen lamp was illuminated for four seconds at the end of each trial (i.e., during the inter-trial interval).

### Onset saccade condition

**Figure 1:**
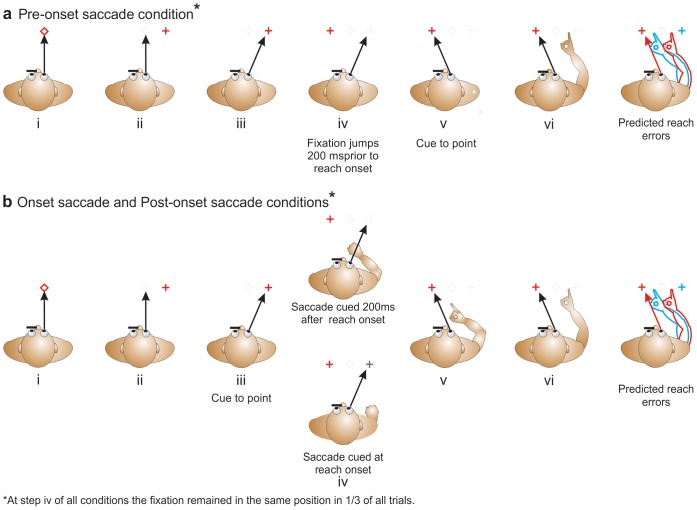
Sequence of events for trials in all conditions. a: *Pre-onset saccade* condition. Participants foveate a target (displayed for 1sec; panel i), before saccading to an eccentric fixation cross which appears as the target disappears (panel iii) and then cued to reach 1s later. 200ms *before* the cue to point the fixation cross jumps to the opposite side of the target location in two thirds of all trials (panel iv). Participants are then cued to point (panel v) and they point with their finger and arm fully extended in complete darkness to the remembered location of the target (panel vi). b: *Onset saccade* and *Post-onset saccade* conditions. In these experimental tasks the second fixation appears either *at* reach onset or 200ms *after* reach onset (panel iv). The eyes then change position in two thirds of all trials while the hand is in flight (panel v). If the fixation cross jumps it shifts to the opposite side of the target site to elicit a gaze-dependent pattern of errors/overshoots [4]. Participants then point as described above (panel vi). Predicted reaching direction if the target is updated as function of the shift in gaze cued at or after reach onset is depicted by the red arm (right panels), and the blue arm represents predicted reaching direction if the remembered target is *not* updated once the reach has begun.

Participants looked at the pointing target (diamond) which was presented for one second (Fig. 1bi) at one of the three locations, followed by the appearance of a fixation-cross (Fig. 1bii) eliciting a saccade to that fixation location as the target disappeared. One second later, participants received an auditory cue to point to the remembered location of the target (diamond; Fig. 1biii). In two thirds of trials this fixation cross jumped to the opposite side of the remembered target location at movement onset (i.e., when the finger left the mouse; Fig. 1biv) eliciting a second saccade to this location while the arm was mid-flight (Fig. 1bv; i.e., participants performed a second saccade to keep their gaze directed at the fixation cross). In the remaining 33% of trials, the fixation cross did not move, and thus gaze remained in the same initial direction for the entire reaching movement. Participants pointed to the remembered target location (Fig. 1bvi), and the trial ended when they returned their hand to the mouse. The combination of targets and crosses were randomized across trials. The central target was combined with all five fixation sites – the 5° targets were combined with only four of them, so that the maximum retinal eccentricity was 15° from center. These 19 target-fixation combinations were repeated 14 times for a total of 266 trials. To reduce fatigue, this condition was split in two sessions (each with 7 repetitions of each possible combination for a total of 133 trials) run on separate days.

### Post-onset saccade condition

Given the results of the above condition (see *Results* below), we wanted to test both whether eye-centered remapping would persist later into the reach movement and also how tightly coupled this updating is to the motor output (i.e., to determine if or when gaze centered updating would no longer be possible, and if the motor output is more influenced by the IGD (i.e., initial target-relative gaze direction) when the saccade is cued later in the reach – that is FGD (i.e., final target-relative gaze direction) should have less of an effect as saccades occur later).

This task was identical to the *Onset saccade condition*, but in this task the cue to perform the second saccade was given not at reach onset, but 200ms post-reach-onset (Fig. 1biv). Again participants pointed to the remembered target location with their arm and index finger fully extended (Fig. 1bvi), and the trial ended when they returned their hand to the mouse. The number of fixation-target combinations, and trials were as above but collected in a single session of 133 trials.

### Pre-onset saccade condition

This task replicated the standard findings of previous work and served as a control to compare how well subjects updated the location of the remembered target following a single versus double saccade prior to reaching. Thus, this condition (Fig. 1ai – v) was identical to those described above except that in two thirds of trials the fixation cross jumped to the opposite side of the remembered target location eliciting a second saccade 200ms *before* the cue to point was given. The number of fixation-target combinations, trials, and sessions were as in the *Onset saccade condition* above.

### Target representation reliability experiment

**Figure 2:**
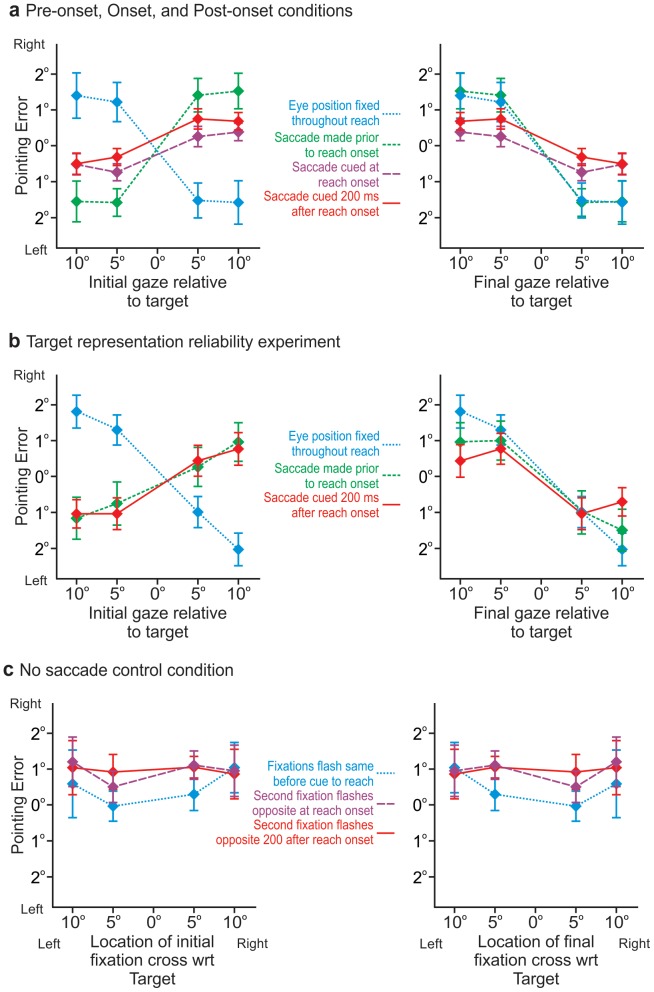
Group mean reach endpoint errors. a: Group mean horizontal pointing errors as a function of target-relative gaze direction for IGD (left panel) and for FGD (right panel). The blue curve represents errors when the eyes remain fixed after moving once following initial foveation of the target (i.e., IGD  =  FGD; ⅓ of all trials in each condition. The green curve represents reach errors when the eyes change position again 200ms *before* the point cue. The purple and red curves represent the errors made when the second saccade is cued *at* reach onset or 200ms *after* reach onset respectively – these errors are the same in magnitude and direction and do not differ from the green curve. b: Group mean horizontal pointing errors as a function of target-relative gaze direction for IGD (left panel) and for FGD (right panel) from the *Target representation reliability experiment*. Blue, green, red curves are as in Figure 2a. c: Horizontal pointing error from the *No saccade control* condition. When the fixation cross appeared and stayed stationary (blue) or jumped to the opposite side of the remembered target location at reach onset (green) or 200ms after reach onset (red), but subjects continued fixating the location where the target had been displayed rather than saccading to the fixation crosses, reaches were unaffected. Error bars in panels a-c represent SEM.

Since the magnitude of reach endpoint errors in the trials where the eyes moved during the reach appeared smaller (although not significantly different; see *Results* below) in the *Onset saccade* and *Post-onset saccade conditions* (Fig. 2a), we conducted an additional experiment to explore this further. Specifically, we wished to investigate whether variability in the *Onset saccade* and *Post-onset saccade* reaching data could be predicted by assuming that reach errors in this condition were due to some combined contribution of IGD and FGD errors. If reaches in the *Onset* and *Post-onset* saccade conditions were generated by combining the pre-saccadic eye-centered representation of target location with a saccade-updated eye-centered representation, then this might explain the reduced endpoint errors. Given that an updated representation would necessarily be derived from the non-updated pre-saccade representation, and given that updating is likely noisy, a combination of the pre-saccade and post-saccade representations should yield larger reaching variance in these conditions than in conditions with no updating or where updating was completed before reach onset (see *Discussion* for further details).

This additional experiment allowed us to collect a dataset suitable for generating variability predictions that were not possible with our existing data by 1) ensuring that the two fixation directions differed not only in terms of relative direction of the remembered target location, but also in magnitude; and 2) allowing us to collect a sufficient number of trials to achieve a reasonable estimate of variability – the previous conditions did not include enough trials to allow us to do this.

Apparatus, stimuli, and all other parameters were the same in this experiment as in the conditions of the previous experiments. Here participants either completed a single saccade prior to reach onset and maintained fixation at this location throughout reach, or they made a second saccade 200ms *before* the cue to reach, or 200ms *after* reach onset as in the previous conditions. In conditions with a second saccade, FGD was on the opposite side of the target location compared with IGD, and was of larger or smaller eccentricity relative to the target. We collected 42 repetitions of each possible target and fixation combination, and 48 trails where the fixation location was the same as the target (as a control) for a total of 624 trials that were collected in three sessions of 208 trials each on separate days.

### No saccade control condition

Given the results of the above testing conditions (see *Results* below) we wanted to rule out the possibility that the effects we had found were simply due to attention or distracter effects of the flashing fixation stimuli.

The structure of the stimuli presentation was the same as other conditions, but participants maintained fixation at the remembered target location throughout the trial, ignoring both fixation crosses and pointing normally to the remembered location of the target (i.e., where they were looking). That is, instead of saccading to the two flashed fixation crosses participants maintained their gaze at the remembered location of the target as the first cross flashed when the target disappeared (after 1s of foveating the target), and as the second cross flashed (either at reach onset, or 200ms following reach onset). This condition was collected as a single session of 160 trials.

### Data processing and analysis

Kinematic data of the eye, head and arm were exported from the MotionMonitor, and combined with the command file of the laser projector allowing the data to be temporally aligned with the appropriate stimulus combination. These integrated files were then viewed in a graphical user interface (GUI) custom developed and executed in MatLab 7.1 (The MathWorks Inc., Natick, MA).

Data points of the arm and eye recordings were then manually selected in the GUI at nine (9) separate event markers. These included: 1) when the pointing target was displayed; 2) when the fixation-cross was displayed; 3) during stable pointing (i.e., when maximal amplitude was reached and velocity *appeared* to be 0 mm/s in the GUI; Cf., [12], [14]; 4-5) at movement onset and offset of the pointing movement; and 6–9) at movement onset and offset of both saccades. Trials in which the eye moved inappropriately (i.e., at the wrong time or to the wrong location) were discarded. A custom MatLab routine was then used to identify potential gaze or arm outliers (±2 SD the respective mean). Each trial identified as an outlier was then inspected to determine if they were indeed mistrials to be removed from analysis of if they were identified as outliers due to a mis-selection of one or more data points (data removed as outliers accounted for approximately 6% of all data collected). As in our previous studies, pointing errors for each movement were calculated by subtracting the finger position during pointing from those in baseline testing conducted at the conclusion of each block of trials (i.e., normal pointing to the five fixation locations with full vision of the arm, target, and surrounding environment). We were specifically interested in horizontal angular errors as a function of horizontal movement of the eyes. When saccades were made during the reaches we were also interested in reach trajectory deviations relative to saccade onset and offset.

In order to analyse reach trajectories and determine path deviation relative to reach completion we temporally normalised the data (to account for differing movement times between reaches and subjects; ∼572 ± 91ms on average across subjects and conditions) by dividing each trial into 50 samples (each sample taken at intervals of 2% of movement completion). Custom MatLab routines were then used to plot the horizontal displacements of the finger tip, and mark movement onset and offset of the eye during the reach when applicable. The mean reach trajectories for each subject for each target-relative gaze direction were then compared to trajectories of reaches to targets when the eye did not move (but began with the same initial target-relative gaze deviation) to ascertain the location at which the reach trajectories from the former significantly deviated from the latter.

Since the finger began at approximately the same position for every trial we assumed that these two discrete time-sampled trajectories coincided for a portion of the movement. If the change in gaze direction with respect to the target has an effect on reach endpoints then these trajectories should subsequently diverge following the saccade. So, we had to compare these trajectories to detect the time of this divergence. This leads to a number of statistical issues.

Performing such a comparison with t-tests for instance leads to two related problems. First, as many comparisons as time points (or epochs/bins) are required, leading to an enormous reduction in power if these tests are corrected with typical multiple comparison procedures. Thus, employing corrected t-tests would lead to a very late time of divergence, or even failure to detect a divergence at all. Second, ignoring the multiple comparison issue would lead to numerous Type I errors, possibly detecting a divergence that does not exist. Moreover, following the trajectory in time, the first of these Type I errors is quite likely to occur before the actual time of divergence (if it exists), but by a completely unpredictable amount. Thus, the t-test is a poor criterion because it will almost certainly provide a result that is too early (uncorrected), or too late (corrected).

Empirically, people tend to move along trajectories that minimize jerk [25]. So we hypothesize that an “in-flight” or “online” correction of a hand trajectory would cause a deviation from minimum jerk, likely at the point of correction. We can test this by comparing peak jerk magnitude in reaches during which the eyes did not move mid-transport, and peak jerk magnitude of reaches during which the eyes did move during the reach. Peak jerk should be higher in the latter. If this is the case, then the time of this peak jerk should correspond, at least approximately, to the time of course correction.

Calculating third derivatives directly from a trajectory sampled at discrete time intervals would lead to large errors. Instead, for each spatial component of a position sample taken at a particular time, t, we fit a quartic (i.e., fourth order) polynomial to that point, (x(t), y(t), z(t)), and its ten temporally nearest neighbors (i.e., the five position samples immediately before and the five immediately after that point). Third order derivatives could then be calculated analytically from the three resulting polynomials, providing the three jerk components at t. Of course, this procedure could not provide estimates for the first or last five time points of the trajectory, which is not problematic since path deviations during this time period are highly unlikely.

### Data analysis

*Pre-onset saccade*, *Onset saccade*, and *Post-onset saccade conditions* were evaluated separately using repeated measures analysis of variance (RM-ANOVA) in IBM SPSS Statistics 20 with the factors: 2 (fixation jump: fixation remained stationary or fixation jumped) by 4 (target-relative gaze direction: –10, –5, 5, 10). Statistical evaluation of the *Target representation reliability experiment* was conducted in the same manner. The *No saccade control* condition was evaluated with an RM-ANOVA of: 3 (fixation jump: fixation remained stationary, fixation jumped at reach onset, or fixation jumped 200ms after reach onset) by 4 (fixation relative to target: –10, –5, 5, 10). Omnibus comparisons between conditions and experiments were conducted using mixed RM-ANOVAs structured as above, including a between subjects factor of “Condition” (3: pre-reach saccade cue, reach onset saccade cue, post-onset saccade cue) and/or “Experiment” (2: main experiment, target representation reliability experiment) where necessary. All effects were evaluated with an alpha level of 0.05. Appropriate post-hoc comparison procedures were used to further explore significant main effects (e.g., Tukey’s HSD; other post-hoc comparisons specified below where appropriate) and interactions (i.e., simple-effects ANOVA followed by Tukey’s HSD) as necessary.

The velocity profiles of the eye and hand were also evaluated to determine if the pointing movements were disrupted by the mid-reach saccade. All velocity profiles have no apparent deviation regardless of the eye moving during the reach or not, and statistically the same in terms of peak velocity and relative time-to-peak velocity.

## Results

### Reach endpoints

#### No second saccade control trials and Pre-onset saccade conditions

When the eyes move only once after foveating the target and then remain fixed throughout the reach (i.e., IGD  =  FGD as in ⅓ of all trials from all conditions; Blue curves Figures 2a & b) we see a significant and systematic modulation of reach error as a function of target-relative gaze direction consistent with the retinal magnification effect – directing gaze to the left of target site results in rightward error and *vice versa* as above (Cf., [11]) – [F (3,24)  = 21.694; p = 0.0001]; i.e., in a simple effects analysis of only trials where the eyes remain fixed in all conditions there is a main effect of target-relative gaze direction. This finding is not surprising and consistent with the previous studies referred to above.

We have plotted the reach endpoint errors (Figure 2a) as a function of IGD (left panel) and separately as a function of FGD (right panel) to illustrate that the pattern of errors is opposite when IGD ≠ FGD than when IGD  =  FGD. But, note that the control trials where there is no second saccade (Blue curve in Fig. 2a) and trials when the second saccade occurs prior to reach onset (Green curve in Fig. 2a), are plotted as a function of FGD (right panel) that the curves overlap and there is no significant difference between these gaze-dependent errors (the curves are identical; [F (3,24)  = 0.254; p = 0.858]; i.e., there is no interaction effect between number of saccades (i.e., one versus two saccades; or IGD  =  FGD versus IGD ≠ FGD) and final target-relative gaze direction. As expected the modulation of reach error as a function of FGD in the *Pre-onset saccade condition* is also significant [F (3,24)  = 15.68; p = 0.0001]. Again, finding gaze dependent error following multiple saccades prior to reach onset is not surprising [12], and demonstrates that the paradigm is eliciting the expected response.

#### Onset saccade, and Post-onset saccade conditions

Interestingly, the shift in the direction of reach errors not only occurred when the eyes moved to the opposite side of the target well before reach onset (i.e., IGD ≠ FGD), but also when the eyes moves during reaching movement. In both conditions where a second saccade is performed during the reaching movement (Red and Purple curves Fig. 2a), we also see that the pattern of reach errors also varies systematically with FGD as in the *Pre-onset saccade condition* (Green curve). This modulation of reach error as a function of FGD is significant in both of these conditions as well [*Onset saccade*: F (3,24)  = 9.311; p = 0.0001; and *Post-onset saccade*: F (3,24)  = 8.622; p = 0.019].

Although these curves (Red and Purple curves Fig. 2a) do not overlap perfectly with the *Pre-onset saccade condition* (Green curve Fig. 2a), the differences between the three curves do not achieve statistical significance [F (3,24)  = 2.028; p = 0.137]; i.e., there is no interaction effect between condition and final target relative gaze direction. Further, none of these curves significantly differ from the Blue curve which represents the other ⅓ of trials in all conditions where there is no second saccade (all comparisons p > 0.05). Thus, all trials for all conditions show the typical overshoot in pointing in the direction opposite to *final* gaze direction, no matter if gaze is directed there prior to reach onset, at reach onset, or 200 ms after reach onset. The saccade-dependent deviations in the reach trajectory, and *when* they occur, are discussed in the *Reach trajectories* section below.

#### Target representation reliability experiment

Given that the modulation of reach errors as a function of FGD for the *Onset* and *Post-onset saccade* reaching conditions appeared smaller (although not significantly so) than in those trials when the eyes did not move during the reach, we designed this second experiment to test if this “reduction” in the gaze-dependent reaching errors might have been due to some combined contribution of IGD and FGD that would be evidenced by variability in the data. Figure 2b shows that in this experiment, as in the previous experiment, we again find a significant effect of gaze on reach error in all conditions [*Single saccade control trials*: F (3,21)  = 9.696; p = 0.0001; *Pre-onset saccade*: F (3,21)  = 4.506; p = 0.014; and *Post-onset saccade*: F (3,21)  = 6.751; p = 0.002] and again we find that the magnitude in reach error appears to be reduced when the second saccade is cued 200ms *after* reach onset. Unlike in the previous experiment we also find that the magnitude of the pattern of reach errors appears smaller overall when the second saccade is cued 200ms *before* the cue to reach. However, there is again no statistically significant difference between the conditions as a function of FGD. As predicted, reach endpoint variability significantly differs across the three experimental conditions (*Single saccade*, *Pre-onset saccade*, or *Post-onset saccade*; [F(2,14)  = 6.74; p = 0.009]). If the reaching errors that we observe here are due to some combined influence of IGD and FGD, then the variance between conditions should differ most greatly between the *Single saccade* and *Post-onset saccade* conditions. Comparing variance (i.e., standard deviations of reach endpoints) between the two conditions where we would have expected there to be the greatest difference in variance if there was indeed a combined contribution of IGD and FGD (i.e., *Single saccade* v. *Post-onset saccade*), we find no significant difference (p =  0.331). Furthermore, post-hoc tests (Bonferroni) reveal no significant differences between any of the conditions (*Single saccade* v. *Pre-onset saccade*: p =  0.104; *Single saccade* v. *Post-onset saccade*: p =  0.994; *Pre-onset saccade* v. *Post-onset saccade*: p =  0.06). Thus, the internal representation of the remembered target derived from FGD does not seem to be combined with some prior target representation resulting from IGD.

We compared the data from this experiment with the conditions of the original experiment using RM-ANOVA with “condition” as a between-subjects factor, and find that reach endpoints from all conditions across the two experiments are not significantly different, neither in terms of the direction and magnitude of reach error [F (6,42)  = 1.137; p =  0.348] nor the precision of reach error [F (6,42)  = 1.207; p = 0.398].

#### No saccade control condition

This condition was conducted to determine if the effects we found above were simply due to attention or distracter effects of the flashing fixation stimuli. We find no significant variation or modulation in reach endpoint error (Fig. 2c), as a function of when the crosses appeared [F (2,6)  = 0.964; p = 0.433], or where they appeared relative to the target [F (3,9)  = 0.062; p = 0.978]. These findings indicate that the flashing of the fixation crosses during the reaches had no distracter or attention effects. We are not aware of another attentional mechanism that may have led to the results that we have found.

### Index of agreement

**Figure 3:**
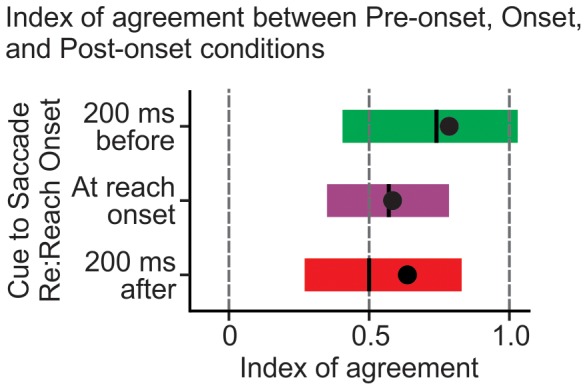
Index of Agreement. Indices representing a comparison of reach errors produced following a second saccade, against those produced when the eyes had been fixed at either the same location as the start (IGD) of that second saccade or fixed at the end (FGD) of that second saccade. This index effectively depicts the agreement of the curves in Figure 2a as a function of IGD versus as a function of FGD. An index of 1.0 represents complete agreement between the curves as a function of FGD, thus representing eye-centered target remapping solely as a function of FGD (i.e., complete and continuous online remapping) – an index of 0 represents complete agreement between the curves as a function of IGD, thus representing eye-centered updating that depends exclusively on IGD (i.e., no online remapping). Vertical black lines represent the median values, the black circles the means, and the shaded boxes 68% CI. In all cases the bulk of the data falls closer to 1 than 0. This suggests continuous eye-centered target updating.

To quantify the degree to which reach endpoint error could be predicted as a function of IGD versus a function of FGD, we developed an index (Fig. 3). The index considers reach error in each condition as a function of IGD and as a function of FGD against the reach error for trials when IGD  =  FGD. By considering the differences in the reach errors of each condition and the control trials the index reflects the level of agreement between the two. Here an index of 0 represents perfect agreement between the reach errors of a given condition and the control trials when expressed as a function of IGD, and an index of 1.0 represents perfect agreement between the reach errors of a given condition and control trials when expressed as a function of FGD. Thus, an index of 1.0 represents perfect agreement between our data and a model of complete eye-centered updating continuously throughout reaching, and an index of 0 represents no eye-centered updating after the first saccade.

In all conditions the indices are significantly greater than 0.5 [*Pre-onset saccade*: t(8)  = 4.542; p = 0.002; *Onset saccade*: t(8)  = 3.543; p = 0.008; *Post-onset saccade:* t(8)  = 2.607; p  = .031]. It is again clear in Figure 3 in all conditions, as in Figures 2a & b, that the patterns of gaze-dependent reach errors across our double saccade conditions tend to be more indicative of continuous eye-centred updating than they are indicative of there being no eye-centred updating. This higher level of agreement in the data as a function of FGD suggests that the representation of the remembered target is updated as a function of current target-relative gaze direction, even while that gaze direction changes during a reaching movement. Finding indices less than 1.0 (i.e. lack of perfect agreement with a model in which FGD determines reach endpoint error) would not be unexpected in this experiment since motor output can be influenced by predicted states generated from previous representations [26]. This seems somewhat contradictory to our finding above that the internal representation of the remembered target derived from FGD is not combined with some prior target representation resulting from IGD. Indeed, less conservative post-hoc tests (Fisher’s LSD) do reveal that there are significant differences in variance in some conditions (*Single saccade* v. *Pre-onset saccade*: p =  0.035; *Pre-onset saccade* v. *Post-onset saccade*: p =  0.02). So, there may be some influence of a prior derived from IGD (or some other body-centred reference frame), but it is clear that reach error is more dependent of the representation derived from FGD, and these data suggest online eye-centred updating (i.e., there is no indication that a model based on no eye-centred updating at all would stand).

### Reach trajectories

**Figure 4:**
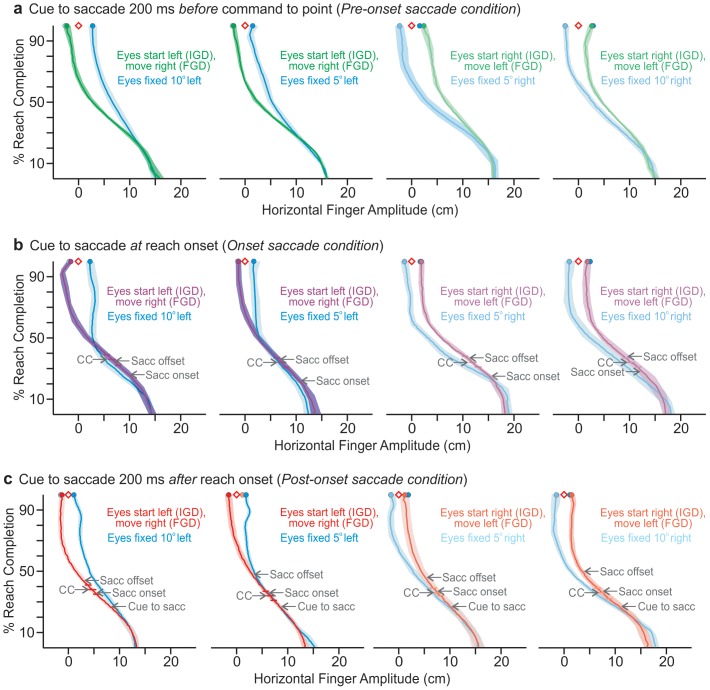
Above view of group mean fingertip trajectories for each condition. Shaded regions represent ±2SEM, horizontal bars indicate occurrence of mean peak jerk (with 95% CI) reflecting course correction (CC), and saccadic onsets and offsets are marked. Horizontal fingertip trajectory is plotted as a function of percent reach completion. The diamonds represent the remembered target locations, and the filled circles represent reach endpoint. Colours represent conditions as in Figure 2a. In all conditions reach endpoint error when the eyes shift is opposite to when the eyes remain fixed. This is true regardless of if the second saccade occurs 200ms before the command to point as in a, or if the saccade concludes as late as 50% of reach completion as in d. Minor differences in start position reflect variation in finger-on-mouse placement and/or the position of the mouse on the table.

Analysis of the reach trajectories (Fig. 4) reveals that the magnitude of peak jerk is significantly greater when a saccade is made than when the eyes remained fixed. This is the case whether the cue to saccade is given *at* reach onset (Fig. 4b), or if it is given 200ms *after* reach onset (Fig. 4c), [t(8)  = 2.4; p<0.05; t(8) = 2.93; p<0.01 respectively]. But this is not the case when the cue to saccade is given 200ms *before* the cue to reach (Fig. 4a; [t(8)  = –0.15; p > 0.88]). The significantly greater magnitude of peak jerk only in those trials when the eyes move during the reach suggests a course correction in the trajectory as a result of the eye movement (the timing is indicated by CC in Figs. 3b-c).

When the cue to saccade was given 200 ms before the command to reach, there was no significant difference between either the magnitude of peak jerk [t(8)  = –0.15; p > 0.88] or time to peak jerk of the reach movement [t(8)  = 1.35; p > 0.21] between trials where the eyes remained fixed and when the eyes jumped. When the cue to saccade was given at reach onset, the time to peak jerk did not differ between trials where the eyes moved, and trials where the eyes remained fixed [t(8)  = 1.26; p > 0.25]. But in the trials where a second saccade was made the magnitude of peak jerk was significantly greater than when the eyes remained fixed [t(8)  = 2.4; p<0.05]. This indicates there was a correction made to the trajectory as the result of the eye movement. When the cue to saccade was given 200 ms after reach onset and a saccade was performed, both time to peak jerk [t(8)  = 10.86; p<0.001] and the magnitude of peak jerk [t(8)  = 2.93; p<0.01], were significantly greater than when the eyes remained fixed. This again suggests that there was a corresponding correction to the trajectory of the hand when the eyes moved. Table 1 indicates the times at which peak jerk occurred during the normalized time it took the hand to reach its final position. It should be noted that while it appears that there may be some coupling between time to peak jerk and the saccadic onset, there is no statistical correlation between path deviation and any recorded event marker.

**Table 1:** Percentage of reach movement completion for all relevant event markers for each condition.

			Direction of fixation jump			
			Right		Left	
			From 10° Left	From 5° Left	From 5° Right	From 10° Right
Cue to saccade	Pre-reach	Course Correction	29	28	29	29
	*at* reach onset	Saccadic Onset	25	23	25	29
		Saccadic Offset	35	36	38	39
		Course Correction	36	34	34	34
	*after* reach onset	Cue to Saccade	28	28	28	28
		Saccadic Onset	37	38	37	43
		Saccadic Offset	44	49	46	50
		Course Correction	38	34	33	36

Reach endpoint errors are again marked in Figure 4, and it is clear here as in Figures 2a and b, that the pointing overshoot in the opposite direction to *final* gaze characteristic of the retinal magnification effect and eye-centred representation/mapping is present in all trials in all conditions. This is the case no matter if gaze is directed to its final position prior to reach onset, at reach onset, or as late as halfway through the reaching movement (Fig. 4c).

## Discussion

Reach errors vary significantly and systematically with the final target-relative gaze direction even when gaze shifts from an initial eccentricity to the opposite side of the remembered target location *during* the reach. This indicates eye-centered updating of reach targets does not end at movement initiation, but that this saccade-driven change in the internal representation of the goal site occurs even while the reaching movement is inflight. Specifically, when a second saccade from one side of the remembered target location (IGD) to the other (FGD), is cued *at* reach onset, so gaze lands in its final direction when the hand is mid-flight, the resulting reach errors (Purple, Figs.2a & 4b) resemble those when gaze remains in the same direction as FGD, and not those when gaze remains in the same direction as the initial target-relative gaze direction. Likewise, the hand paths and errors produced when gaze shifts during the reach (Purple and Red, Figs. 2a & 4b-c) are similar to those produced in our control (i.e., *Pre-onset saccade*) condition, where the second saccade is performed prior to initiation of the reach (Figs. 2a & 4a, Green). When the eyes are cued to move *at* reach onset and 200ms *after* reach onset the peak jerk of the hand path is significantly greater than when the eyes remain fixed. The instant of peak jerk signifies a deviation in the hand path (i.e., a course correction) that is the result of the eyes moving during the reach. The pattern of errors cannot be attributed to attentional effects (Fig. 2c). These findings suggest that visuospatial memory continues to be updated as a function of the target-relative gaze direction (i.e., in an eye-centred reference frame) even after the reach movement has been initiated, and even when the eye reaches its final gaze direction when the reaching movement is more than half completed (Figs. 4b-c).

Given that shifting gaze to a new FGD during reaching has a significant effect on endpoint errors, it is not surprising that the magnitude of peak jerk is significantly greater when a saccade is made than when the eyes remained fixed, whether the cue to saccade is given *at* reach onset, or if it is given 200ms *after* reach onset. But this is not the case when the cue to saccade is given 200ms *before* the cue to reach. Of course, when the eyes move before the reach, there should be no course correction (and thus no significant difference in peak jerk) since in this condition the eye was already at FGD at the time of reach initiation. Yet, there appears to be no meaningful correlation between the timing of course corrections in saccade trials and actual saccade timing (corrections seem to occur at similar times in both *Onset saccade* and *Post-onset saccade conditions*; Table 1). So, while we cannot say specifically *when* the updated target-relative gaze direction is incorporated with the movement plan, it is clear that the intervening eye movements lead to corrections in reach trajectories (Figs. 4b-c; as well as endpoints, Fig. 2a-b), even when gaze shifts well after the reach is initiated. These course corrections are in the direction expected if the targets are remapped as a function of the resulting FGD.

It might be reasonable to suggest that the magnitudes of the reach errors in the *Onset saccade* and *Post-onset saccade conditions* were smaller because the initial, pre-saccadic internal representation of target location (i.e., from IGD) was being combined with the second and final internal representation of target location (i.e., from FGD). Effectively, the representation of the target from IGD may be acting as a prior and influencing the representation resulting from FGD. By these means the magnitude of reach error would fall between where they would fall if they were influenced only by the non-updated pre-saccadic IGD representation, or only by the post-saccadic FGD representation. That is, the influence of FGD would draw the reach error in one direction, but the remaining influence of IGD (through combination of IGD and FGD) would continue to draw reach error in the opposite direction, such that reach errors might appear to be reduced following the second saccade. Any putative combination of target location representations derived from IGD and FGD would likely be approximately linear. However, the FGD representation would have to be derived *from* the IGD representation via updating. Thus, noise in the FGD representation would be identical to that in the IGD representation, plus noise from updating. Any linear combination of IGD and FGD representations should then be *more* variable than the initial IGD representation. Under the assumption of such combination, reaching responses should also be more variable in the experiments with saccades after target offset (IGD ≠ FGD) than in those without (IGD = FGD). But, we do not find this. Therefore, our findings are clearly incompatible with a model that assumes linear combination of IGD and FGD representations of target location. If it were the case that updating was a noise-free process so the combination of IGD and FGD resulted in no additional noise being introduced, then we should see no effect of gaze modulation on reach error (i.e., the effects of FGD and IGD on reach error would wash each other out and we would not find significant modulation of error with FGD). This is not the case (Fig. 2b). Although we cannot rule out some combination of IGD and FGD, it is unclear what sort of combination could produce the data we have, and the apparent indication that IGD and FGD are not combined, is not due to a lack of power (Observed Power  =  0.845). Thus, the reduced retinal magnification magnitude, in trials with a second saccade, is likely due to a processing delay in incorporating current target-relative gaze direction with the reach plan (while updated representations may be incorporated in the movement plan until the reach is completed, this processing delay likely means that the reach trajectory and endpoints would not reflect this if saccades were initiated much later than 200 ms after reach initiation - as mentioned above we cannot say exactly when an updated representation would be incorporated with the movement plan). This influence of processing delay can explain why the magnitude of gaze-dependent errors for the *Onset saccade* and *Post-onset saccade conditions* (Red and Purple curves Fig. 2a) looks smaller (although it is not significantly smaller) than that for the *Pre-onset saccade condition* (Green, Fig. 2a). The same logic and explanation can be applied to the apparently, yet not significantly, smaller modulations depicted in Figure 2b.

Remembered target representations are remapped in an eye-centered reference frame when the eyes change direction even while the hand is in mid-transport. Therefore representations in this frame must persist in parallel with other representations even well after the movement is initiated. Eye-centered representations do not only occur in early stages of sensori-to-motor transformation as shown previously in neurophysiological studies of reach planning [15]–[20], but contribute continuously and dynamically to online motor control.
